# Plasma VEGF Concentrations and Ketamine's Effects on Suicidal Ideation in Depression With Suicidal Ideation

**DOI:** 10.3389/fpsyt.2022.855995

**Published:** 2022-04-25

**Authors:** Wei Zheng, Li-Mei Gu, Yan-Ling Zhou, Cheng-Yu Wang, Xiao-Feng Lan, Bin Zhang, Yu-Ping Ning

**Affiliations:** ^1^The Affiliated Brain Hospital of Guangzhou Medical University, Guangzhou, China; ^2^The First School of Clinical Medicine, Southern Medical University, Guangzhou, China

**Keywords:** ketamine, VEGF, suicidal ideation, depression, response

## Abstract

**Objectives:**

Accumulating evidence supports a role for vascular endothelial growth factor (VEGF) in the pathogenesis of depression, but its relationship with the antisuicidal effects of ketamine is not clear. Our objective was to determine whether there was an association between the plasma VEGF (pVEGF) concentrations and the antisuicidal response to serial ketamine infusions.

**Methods:**

Six ketamine infusions (0.5 mg/kg) over a 12-day period were administered to sixty depressed individuals suffering from suicidal ideation. The Hamilton Depression Rating Scale (HAMD) suicide item, the Montgomery-Åsberg Depression Rating Scale (MADRS) suicide item, and the Beck Scale for Suicide Ideation (SSI-part I) were used to assess suicidal ideation at baseline, 1 day after the first infusion (day 1), 1 day following the last infusion (day 13), and again 2 weeks post-infusion (day 26). For this purpose, plasma was obtained at baseline, day 13 and 26.

**Results:**

The rates of antisuicidal response to ketamine were 61.7% (37/60), 81.7% (49/60), and 73.3% (44/60) at days 1, 13, and 26, respectively. The linear mixed model revealed significant time effects on suicidal ideation and pVEGF concentrations over time (all *Ps* < 0.05). Antisuicidal responders did not have significantly altered pVEGF concentrations compared with non-responders on day 13 and day 26 (all *Ps* > 0.05). No significant correlation was found between the baseline pVEGF concentration and suicidal ideation as measured by the SSI part 1, HAMD suicide item and MADRS suicide item on days 1, 13, and 26 (all *ps* > 0.05).

**Conclusion:**

This preliminary finding does not support a role for VEGF in the antisuicidal effects of serial ketamine treatments in individuals with depression and suicidal ideation. Further research is needed to confirm and expand these findings.

## Introduction

Approximately 0.8 million individuals worldwide die by suicide every year (1), which is becoming a substantial public health concern. Suicide is a complex and multifaceted phenomenon where numerous potential mechanisms could be implicated (2). Suicidal ideation is common in individuals with major depressive disorder (MDD) (3) and bipolar depression (BD) (4), especially among inpatients. Better therapy for suicidal ideation in MDD and BD is a critical target in preventing deaths due to suicide (5). However, very few treatments can rapidly alleviate suicidal ideation (6). Although accumulating evidence has indicated that treatment with dialectical behavioral therapy (7), cognitive behavioral therapy (CBT) (8, 9), and lithium (10) can effectively alleviate suicidal ideation, the onset of clinically relevant antisuicidal effects generally takes 2–4 weeks. Thus, novel pharmacotherapeutic approaches are urgently needed for subjects with depression and suicidal ideation.

As a non-selective *N*-methyl-D-aspartic acid receptor (NMDAR) antagonist, ketamine has shown quick and dramatic antisuicidal effects in randomized controlled trials (RCTs) (11, 12) and meta-analyses (13, 14) for MDD and BD. In addition to its rapid antisuicidal effects, ketamine at a single intravenous dose has a rapid effect in reducing the level of ahedonia (15–18) and ameliorating depressive symptoms (19, 20) in MDD and BD. After controlling for the effects of ketamine on depression, ketamine's antisuicidal ideation remained significant (13, 21). Antidepressant and antisuicidal responses to a single ketamine infusion could be prolonged with repeated ketamine infusions (22, 23). For example, a recent study found that the antisuicidal response rates increased from 57.0 to 65.1% after five additional infusions of ketamine in depressed patients experiencing suicidal ideation (22). However, a certain proportion of depressed patients experiencing suicidal ideation do not adequately respond to single or repeated ketamine infusions, but the reasons for this are unclear.

Vascular endothelial growth factor (VEGF), as an angiogenic cytokine, has been associated with the antidepressant response to electroconvulsive therapy (ECT) (24) and serotonin selective reuptake inhibitors (SSRIs) (25). Patients experiencing suicidal ideation had lower cerebrospinal fluid VEGF concentrations than healthy controls (26). In contrast, antidepressant therapy can induce hippocampal expression of VEGF (27). Recently, Deyama et al. found that the rapid antidepressant response to ketamine was associated with neuronal VEGF-Flk-1 signaling in the medial prefrontal cortex (mPFC) (28). Finding on the relationship between VEGF and ketamine's antidepressant effect in depressed patients were inconsistent (29, 30). For instance, McGrory et al. found that VEGF plays an essential role in the antidepressant action of ketamine (30). However, another study reported a negative finding (29). No study has yet reported on the association of plasma VEGF (pVEGF) concentrations and the antisuicidal effects of repeated-dose intravenous ketamine in Chinese subjects with depression who are experiencing suicidal ideation.

The aim of the current study was to: (1) detect the change in pVEGF concentrations after repeated-dose intravenous ketamine in depressed patients experiencing suicidal ideation and (2) to demonstrate the relationship between pVEGF concentrations and the antisuicidal effects of repeated doses of intravenous ketamine. We hypothesized that pVEGF concentrations would be increased after six ketamine infusions, and pVEGF would play an improtant role in the antidepressant actions of ketamine in individuals with depression and suicidal ideation.

## Methods

### Study Population and the Procedure

The data for this study were obtained from a single-center clinical trial (Registration Number: ChiCTR-OOC-17012239) in which unipolar and bipolar depressed patients received six ketamine infusions at the Affiliated Brain Hospital of Guangzhou Medical University from September 2016 to December 2017 (31, 32). All patients gave written informed consent and the study was approved by the Affiliated Brain Hospital of Guangzhou Medical University Institutional Review Board (Ethical Application Ref: 2016030). In the present study, we specifically report the association between pVEGF concentration and the effect of ketamine on suicidal ideation, focusing on depressed patients with suicidal ideation. The inclusion criteria were as follows: (1) sixty depressed patients were 18–65 years old with suicidal ideation as defined by the Beck Scale for Suicide Ideation (SSI)-part I ≥ 2 (33, 34); (2) patients fulfilling the diagnostic criteria listed in the DSM-5, for MDD or BD without psychotic symptoms; (3) each participant experiencing a major depressive episode of at least moderate severity, as defined by the 17-item Hamilton Depression Rating Scale (HAMD) ≥ 17 (35, 36); (4) full understanding of the study procedure. The exclusion criteria of the current study were consistent with those used in previous studes (31, 32). Briefly, participants diagnosed with other psychiatric disorders such as schizophrenia, substance use disorder or alcohol use disorder were excluded, but a comorbidity of obsessive compulsive disorder, anxiety disorder or eating disorder was permitted when it was not judged to be the primary presenting problem. All subjects received six intravenous infusions of ketamine at subanaesthetic doses over 12 days. During the study period, the participants continued their psychotropic medications.

### Antisuicidal Response

The SSI part I, the Montgomery-Åsberg Depression Rating Scale (MADRS) suicide item, and the HAMD suicide item were used to evaluate the severity of suicidal ideation at baseline, 1 day after the first infusion (day 1), 1 day after the completion of six ketamine infusions (day 13), and at the 2-week follow-up after the completion of six ketamine infusions (day 26). Antisuicidal responses to repeated-dose intravenous ketamine at day 13 were defined by the SSI part I < 2 (22, 37).

### Measurement of pVEGF Concentrations

A commercially available enzyme-linked immunosorbent assay (ELISA) kit (R&D Systems, Minneapolis, USA) was used to examine the pVEGF concentrations according to the manufacturer's recommendations. The plasma was obtained at baseline and then on days 13 and 26. The measurement of the pVEGF concentrations was in line with those used in a recent study (29).

### Statistical Analysis

Data from this study were analyzed by using SPSS 24.0 statistical software. Significance was considered at *p* < 0.05. The demographic and clinical characteristics and pVEGF concentrations at baseline were compared between the antisuicidal responders and the non-responders using the chi-squared test and/or Fisher's exact test for categorical variables and Student's *t*-test and/or the Mann–Whitney U test for continuous variables, as appropriate. Changes in the pVEGF concentrations and the suicidal symptoms evaluated by the HAMD suicide item, MADRS suicide item, and SSI part I over time and the subgroup differences (antisuicidal responders vs. non-responders) were investigated using linear mixed models. The association of pVEGF concentrations with the antisuicidal effects of ketamine was examined by correlation analysis.

## Results

pVEGF concentrations were obtained from 60 patients suffering from depression and suicidal ideation. A comparison between the demographic and clinical characteristics of antisuicidal responders and non-responders is presented in Table 1. Antisuicidal responders had marginally significantly higher baseline pVEGF concentrations than antisuicidal non-responders (*P* = 0.07; Table 1).

**Table 1:** Baseline characteristics of antisuicidal responders and non-responders calculated by SSI part I scores on day 13.

**Variables**	**Total sample** **(*****n*** **=** **60)**		**Antisuicidal responders** **(*****n*** **=** **49)**		**Antisuicidal non-responders** **(*****n*** **=** **11)**		**Statistics**		
	***N***	**%**	***N***	**%**	***N***	**%**	**X^**2**^**	**df**	***p***
Male	27	45.0	22	44.9	5	45.5	—^a^	—^a^	1.00
Employed	23	38.3	21	42.9	2	18.2	—^a^	—^a^	0.18
Married	34	56.7	27	55.1	7	63.6	—^a^	—^a^	0.74
On ADs two or more	8	13.3	8	16.3	0	0	—^a^	—^a^	0.33
On APs	35	58.3	29	59.2	6	54.5	—^a^	—^a^	1.00
On mood stabilizers	16	26.7	13	26.5	3	27.3	—^a^	—^a^	1.00
On benzodiazepines	27	45.0	21	42.9	6	54.5	—^a^	—^a^	0.52
On anxiolytics	27	45.0	22	44.9	5	45.5	—^a^	—^a^	1.00
On anticholinergics	8	13.3	8	16.3	0	0	—^a^	—^a^	0.33
	**Mean**	**SD**	**Mean**	**SD**	**Mean**	**SD**	**T/Z**	**df**	***p***
Age (years)	35.3	12.4	34.8	12.0	37.4	14.4	−0.6	58	0.54
Education level (years)	12.2	3.5	12.5	3.4	10.7	3.9	0.8	58	0.14
BMI	22.2	3.5	22.1	3.3	22.8	4.5	−0.7	58	0.51
Illness duration (months)	91.5	80.0	81.5	69.1	135.9	110.2	−2.1	58	**0.04**
FLUeq (mg/day)	35.6	20.7	35.8	21.7	34.5	16.2	0.2	58	0.85
CPZeq (mg/day)	169.1	117.6	168.7	125.8	170.8	74.5	−0.5	—^b^	0.64
Baseline pVEGF concentrations (ng/ml)	38.4	55.7	44.5	59.7	11.6	15.7	−1.8	—^b^	0.07
Baseline SSI-part I scores	5.0	2.4	4.8	2.5	5.2	2.2	0.34	58	0.73
Baseline HAMD suicide item scores	2.2	0.8	2.2	0.8	2.5	0.8	−1.5	58	0.15
Baseline MADRS suicide item scores	2.9	1.3	2.8	1.2	3.5	1.5	−1.8	58	0.07

*Bolded values are p < 0.05*.

a *Fisher's Exact Test*.

b *Mann-Whitney U test*.

*Ads, Antidepressants; APs, antipsychotics; BMI, body mass index; CPZeq, chlorpromazine equivalent milligrams; FLUeq, Fluoxetine equivalents equals; pVEGF, plasma vascular endothelial growth factor; df, degrees of freedom; HAMD, the Hamilton Depression Rating Scale; MADRS, the Montgomery-Åsberg Depression Rating Scale; SSI, the Beck Scale for Suicide Ideation; SD, standard deviation; TRD, treatment refractory depression*.

**Figure 1:**
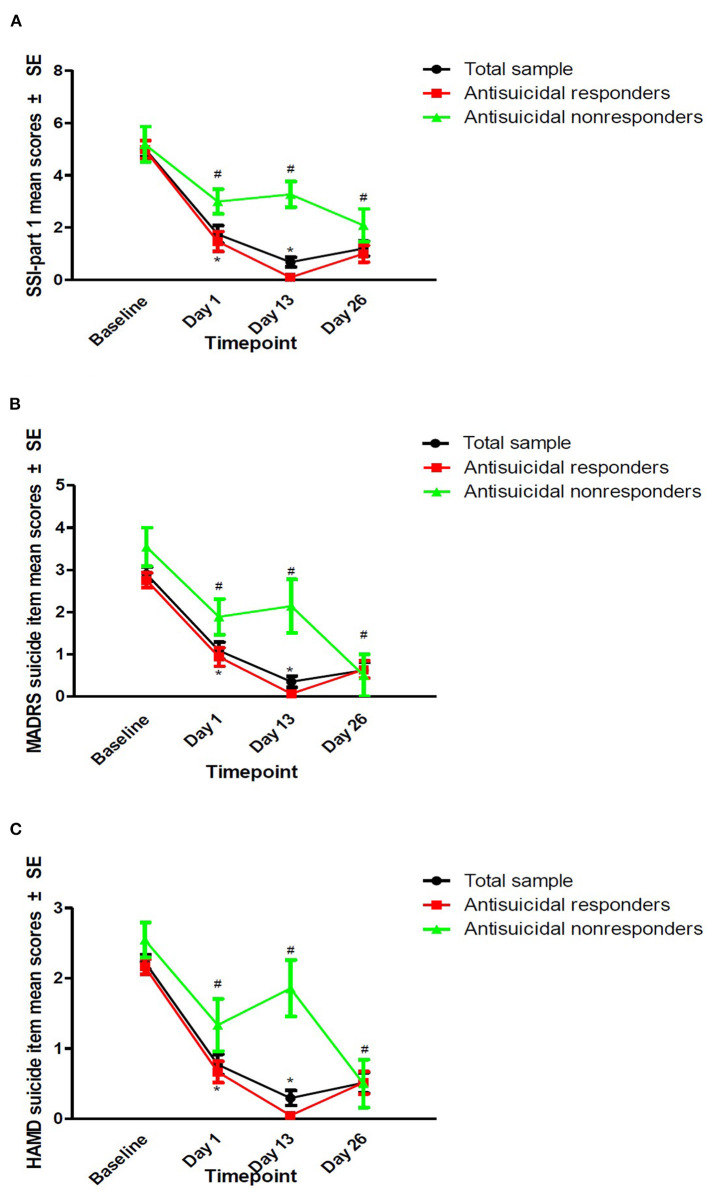
The antisuicidal effects of ketamine were measured by SSI part I, the MADRS suicide item, and the HAMD suicide item. The antisuicidal effects of ketamine as measured by SSI part I **(A)**, the MADRS suicide item **(B)**, and the HAMD suicide item **(C)**. #A significant difference was found compared to the baseline at the indicated times (*P* < 0.05). *A significant difference was found between antisuicidal responders and non-responders at the indicated times (*P* < 0.05). SSI, the Beck Scale for Suicide Ideation; MADRS, the Montgomery-Åsberg Depression Rating Scale; HAMD, the Hamilton Depression Rating Scale.

**Figure 2:**
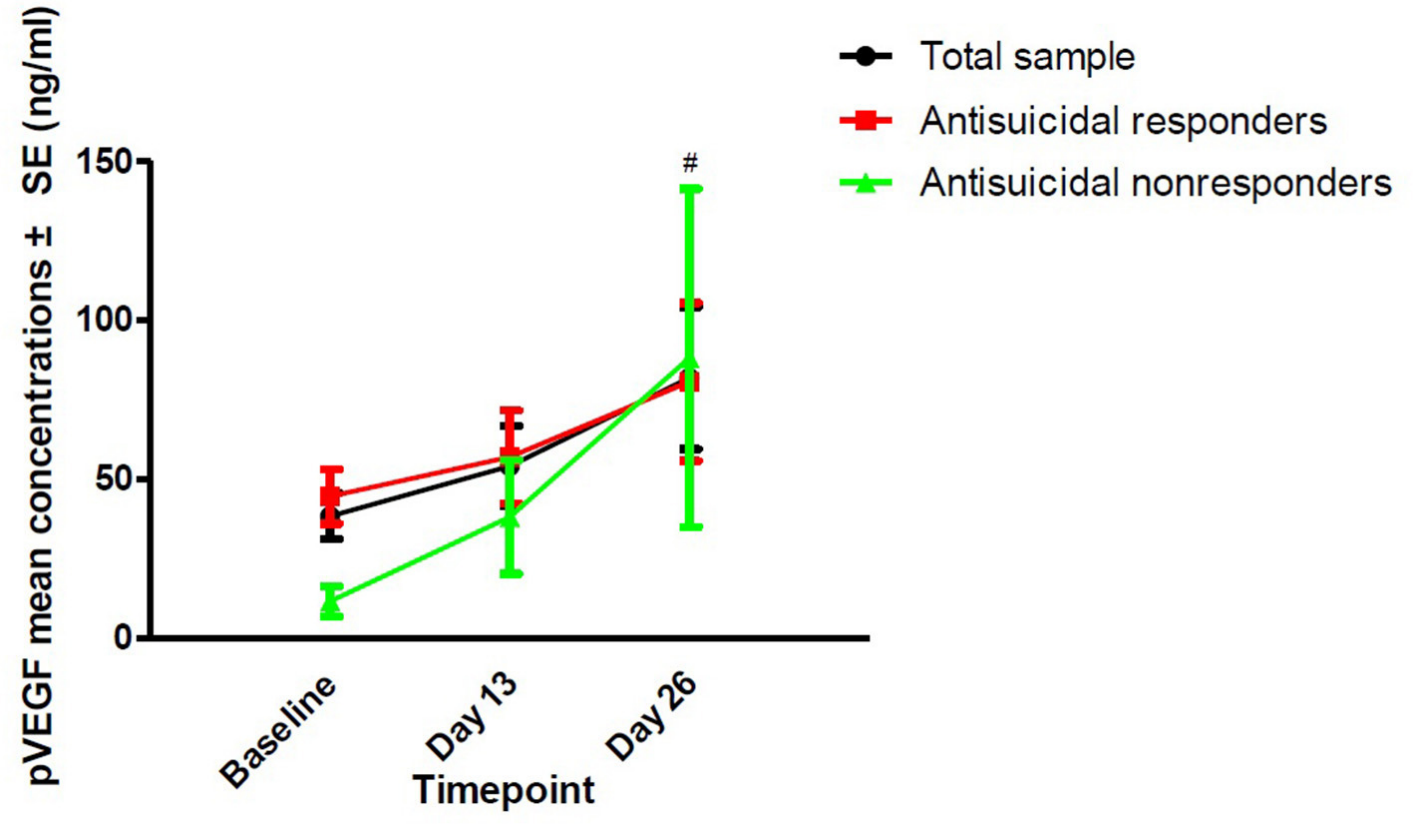
The change in pVEGF concentrations in depressed patients with suicidal ideation at the indicated times. ^#^No significant difference at the indicated times was found when compared to baseline (*P* > 0.05). No significant difference at the indicated times was found between antisuicidal responders and non-responders (*P* > 0.05). pVEGF, plasma vascular endothelial growth factor.

The rates of antisuicidal responses to ketamine were 61.7% (37/60), 81.7% (49/60), and 73.3% (44/60) on days 1, 13, and 26, respectively. The linear mixed model with SSI part I, MADRS suicide items and HAMD suicide items showed a significant main effect of time and group and a group-by-time interaction (all *Ps* < 0.05; Table 2). Antisuicidal responders had a significantly greater reduction in suicidal ideation than non-responders (as measured by the SSI part I, the MADRS suicide item and the HAMD suicide item) at days 1 and 13 (all *Ps* <0.05; Figure 1). The linear mixed model with pVEGF concentrations showed a significant main effect of time (*P* < 0.05; Table 2) but not for the main effect of group and group-by-time interaction (all *Ps* > 0.05; Table 2). Although a significant change in pVEGF concentrations was found at day 26 as compared to baseline (*P* < 0.05), the antisuicidal responders compared to nonresponders did not have significantly altered pVEGF concentrations at day 13 and day 26 (all *Ps* > 0.05; Figure 2).

**Table 2:** Comparison of suicidal ideation scores and pVEGF concentrations between antisuicidal responders and non-responders in depressed patients with suicidal ideation using linear mixed model analysis.

**Variables**	**Group-by-time interaction**		**Time main effect**		**Group main effect**	
	***F***	***p***	***F***	***p***	***F***	***p***
HAMD suicide item scores	6.8	**<0.001**	33.2	**<0.001**	11.9	**0.001**
MADRS suicide item scores	4.6	**0.004**	30.1	**<0.001**	10.2	**0.002**
SSI-part I scores	4.1	**0.008**	28.8	**<0.001**	11.2	**0.002**
pVEGF concentrations (ng/ml)	0.6	0.55	3.5	**0.04**	0.3	0.57

*Bolded values are p < 0.05*.

*pVEGF, plasma vascular endothelial growth factor; SSI, the Beck Scale for Suicide Ideation; MADRS, the Montgomery-Åsberg Depression Rating Scale; HAMD, the Hamilton Depression Rating Scale*.

As depicted in Table 3, no significant association of baseline pVEGF concentrations and reductions in suicidal ideation following ketamine treatment (as measured by the SSI part I, the MADRS suicide item, and the HAMD suicide item) were found on day 1, day 13, or day 26 (all *Ps* > 0.05; Table 2).

**Table 3:** Correlation analysis between suicidal ideation and baseline pVEGF concentrations in depressed patients with suicidal ideation at the indicated times.

**Variables**		**HAMD suicide item scores**			**MADRS suicide item scores**			**SSI-part I scores**		
		**Day 1**	**Day 13**	**Day 26**	**Day 1**	**Day 13**	**Day 26**	**Day 1**	**Day 13**	**Day 26**
Baseline pVEGF concentrations (ng/ml)	*r*	−0.07	−0.18	0.16	−0.05	−0.19	0.13	0.17	−0.22	0.24
	*p*	0.62	0.22	0.30	0.74	0.19	0.41	0.20	0.09	0.07
**Variables**		**Change in HAMD suicide item scores**			**Change in MADRS suicide item scores**			**Change in SSI-part I scores**		
		**Day 1**	**Day 13**	**Day 26**	**Day 1**	**Day 13**	**Day 26**	**Day 1**	**Day 13**	**Day 26**
Change in pVEGF concentrations (ng/ml)	*r*	0.10	−0.14	0.09	0.09	−0.10	0.07	0.10	−0.24	−0.02
	*p*	0.53	0.36	0.54	0.59	0.51	0.67	0.53	0.11	0.91

*pVEGF, plasma vascular endothelial growth factor; r, Pearson coefficient of correlation; SSI, the Beck Scale for Suicide Ideation; MADRS, the Montgomery-Åsberg Depression Rating Scale; HAMD, the Hamilton Depression Rating Scale*.

## Discussion

The current study first illuminated the association of pVEGF concentrations with the effect of ketamine on suicidal ideation. Our findings indicate that antisuicidal responders had marginally significantly greater pVEGF concentrations at baseline than antisuicidal non-responders. Despite a significant reduction in suicidal ideation during serial ketamine infusions over time, the pVEGF concentrations were not significantly altered in antisuicidal responders compared with non-responders on day 13 and day 26. Similarly, no notable association was detected between the pVEGF concentrations and the effects of repeated-dose intravenous ketamine on suicidal ideation as measured by SSI part I, the MADRS suicide item, and the HAMD suicide item.

Accumulating evidence suggests that VEGF is associated with brain function, including neurogenesis, learning and memory, by regulating hippocampal synaptic activity and plasticity (38–41). Dysregulated VEGF concentrations have been involved in major mental disorders, such as MDD and BD (42). Low pVEGF concentrations are associated with a higher suicide risk among suicide attempters (43). Therapy with antidepressants such as SSRIs (27, 44) and ketamine (30) can increase the expression of VEGF. Consistent with previous studies (11–14), in this study, ketamine had a rapid and robust effect in reducing suicidal ideation. Repeated administration of intravenous ketamine (0.5 mg/kg) did not significantly increase pVEGF concentrations, even after a 2-week follow-up, corroborating the results of previous studies (45).

As reported by Deyama et al.'s study, the antidepressant-like and neurotrophic actions of brain-derived neurotrophic factor (BDNF) require VEGF signaling (44). Thus, VEGFR2 signaling appears to be indispensable for cellular and behavioral responses to antidepressant treatments (27). The findings of several animal trials support a role for VEGF in the biological actions of antidepressants (i.e., fluoxetine) (46) and mood stabilizers (i.e., lamotrigine) (47). Similarly, VEGF could mediate the antidepressant actions of electroconvulsive seizures (48, 49) and a single ketamine infusion (28) but not six ketamine infusions (29). However, in this study, we found that VEGF was not involved in the antisuicidal effects of repeated-dose intravenous ketamine in Chinese patients with depression and suicidal ideation, which should be confirmed by RCTs.

This study is associated with several limitations. First, the relatively small sample size is the first study limitation, partly accounting for the negative results. Second, the lack of a control group in the protocol of the present study was another limitation, affecting the interpretation and external validity of the findings. Third, when compared to the samples from controlled clinical trials, the sample of the current study based on a real-world design is potentially more heterogeneous. Furthermore, the pooling of subjects suffering from MDD and BD made the sample non-homogeneous. Fourth, although substance use disorder is a significant predictor of non-adherence among individuals suffering from mood disorders (50), patients suffering from substance use disorder were excluded in this study. Finally, all subjects continued to receive psychotropic medications, which may have potentially affected their pVEGF concentrations and explained the contradictory findings between this study and previous studies (30, 45). Finally, as reported by Levy et al., blood VEGF concentrations may not be associated with VEGF concentrations in the brain (51). However, VEGF concentrations in the brain could not be directly detected in the current study.

## Conclusions

This preliminary study does not support a role for VEGF in the antisuicidal effects of serial ketamine treatments in individuals with depression and suicidal ideation. Further research is needed to confirm and expand these findings.

## Data Availability Statement

The original contributions presented in the study are included in the article/supplementary material, further inquiries can be directed to the corresponding author/s.

## Ethics Statement

The studies involving human participants were reviewed and approved by the Affiliated Brain Hospital of Guangzhou Medical University Institutional Review Board (Ethical Application Ref: 2016030). The patients/participants provided their written informed consent to participate in this study.

## Author Contributions

Y-PN: study design. WZ, Y-LZ, C-YW, and X-FL: data collection. WZ and L-MG: analysis and interpretation of data. WZ: drafting of the manuscript. BZ and Y-PN: critical revision of the manuscript. All authors: approval of the final version for publication.

## Funding

This study was funded by the National Natural Science Foundation of China (82101609), Scientific Research Project of Guangzhou Bureau of Education (202032762), Science and Technology Program Project of Guangzhou (202102020658), the Science and Technology Planning Project of Liwan District of Guangzhou (202004034), Guangzhou Health Science and Technology Project (20211A011045), Guangzhou science and Technology Project of traditional Chinese Medicine and integrated traditional Chinese and Western Medicine (20212A011018), China International Medical Exchange Foundation (Z-2018-35-2002), Guangzhou Clinical Characteristic Technology Project (2019TS67), and Science and Technology Program Project of Guangzhou (202102020658). The funders had no role in study design, data collection and analysis, decision to publish, or preparation of the manuscript.

## Conflict of Interest

The authors declare that the research was conducted in the absence of any commercial or financial relationships that could be construed as a potential conflict of interest.

## Publisher's Note

All claims expressed in this article are solely those of the authors and do not necessarily represent those of their affiliated organizations, or those of the publisher, the editors and the reviewers. Any product that may be evaluated in this article, or claim that may be made by its manufacturer, is not guaranteed or endorsed by the publisher.

## References

[1] Chesney E Goodwin GM Fazel S. Risks of all-cause and suicide mortality in mental disorders: a meta-review. World Psychiatry. (2014) 13:153–60. 10.1002/wps.20128 24890068 PMC4102288

[2] Orsolini L Latini R Pompili M Serafini G Volpe U Vellante F. Understanding the complex of suicide in depression: from research to clinics. Psychiatry Investig. (2020) 17:207–21. 10.30773/pi.2019.0171 32209966 PMC7113180

[3] Cai H Jin Y Liu S Zhang Q Zhang L Cheung T. Prevalence of suicidal ideation and planning in patients with major depressive disorder: a meta-analysis of observation studies. J Affect Disord. (2021) 293:148–58. 10.1016/j.jad.2021.05.115 34192629

[4] Dong M Lu L Zhang L Zhang Q Ungvari GS Ng CH. Prevalence of suicide attempts in bipolar disorder: a systematic review and meta-analysis of observational studies. Epidemiol Psychiatr Sci. (2019) 29:e63. 10.1017/S2045796019000593 31648654 PMC8061290

[5] Ballard ED Wills K Lally N Richards EM Luckenbaugh DA Walls T. Anhedonia as a clinical correlate of suicidal thoughts in clinical ketamine trials. J Affect Disord. (2017) 218:195–200. 10.1016/j.jad.2017.04.057 28477497 PMC5515296

[6] Griffiths JJ Zarate CA Jr. Rasimas JJ. Existing and novel biological therapeutics in suicide prevention. Am J Prev Med. (2014) 47 (3 Suppl. 2):S195–203. 10.1016/j.amepre.2014.06.012 25145739 PMC4143783

[7] Linehan MM Comtois KA Murray AM Brown MZ Gallop RJ Heard HL. Two-year randomized controlled trial and follow-up of dialectical behavior therapy vs therapy by experts for suicidal behaviors and borderline personality disorder. Arch Gen Psychiatry. (2006) 63:757–66. 10.1001/archpsyc.63.7.757 16818865

[8] Hawton K Witt KG Salisbury TLT Arensman E Gunnell D Hazell P. Psychosocial interventions following self-harm in adults: a systematic review and meta-analysis. Lancet Psychiatry. (2016) 3:740–50. 10.1016/S2215-0366(16)30070-0 27422028

[9] Brown GK Ten Have T Henriques GR Xie SX Hollander JE Beck AT. Cognitive therapy for the prevention of suicide attempts: a randomized controlled trial. JAMA. (2005) 294:563–70. 10.1001/jama.294.5.563 16077050

[10] Cipriani A Hawton K Stockton S Geddes JR. Lithium in the prevention of suicide in mood disorders: updated systematic review and meta-analysis. BMJ. (2013) 346:f3646. 10.1136/bmj.f3646 23814104

[11] Price RB Iosifescu DV Murrough JW Chang LC Al Jurdi RK Iqbal SZ. Effects of ketamine on explicit and implicit suicidal cognition: a randomized controlled trial in treatment-resistant depression. Depress Anxiety. (2014) 31:335–43. 10.1002/da.22253 24668760 PMC4112410

[12] Chen MH Lin WC Tu PC Li CT Bai YM Tsai SJ. Antidepressant and antisuicidal effects of ketamine on the functional connectivity of prefrontal cortex-related circuits in treatment-resistant depression: A double-blind, placebo-controlled, randomized, longitudinal resting fMRI study. J Affect Disord. (2019) 259:15–20. 10.1016/j.jad.2019.08.022 31437695

[13] Wilkinson ST Ballard ED Bloch MH Mathew SJ Murrough JW Feder A. The effect of a single dose of intravenous ketamine on suicidal ideation: a systematic review and individual participant data meta-analysis. Am J Psychiatry. (2018) 175:150–8. 10.1176/appi.ajp.2017.17040472 28969441 PMC5794524

[14] Xiong J Lipsitz O Chen-Li D Rosenblat JD Rodrigues NB Carvalho I. The acute antisuicidal effects of single-dose intravenous ketamine and intranasal esketamine in individuals with major depression and bipolar disorders: a systematic review and meta-analysis. J Psychiatr Res. (2021) 134:57–68. 10.1016/j.jpsychires.2020.12.038 33360864

[15] Lally N Nugent AC Luckenbaugh DA Ameli R Roiser JP Zarate CA. Anti-anhedonic effect of ketamine and its neural correlates in treatment-resistant bipolar depression. Transl Psychiatry. (2014) 4:e469. 10.1038/tp.2014.105 25313512 PMC4350513

[16] Lally N Nugent AC Luckenbaugh DA Niciu MJ Roiser JP Zarate CA Jr. Neural correlates of change in major depressive disorder anhedonia following open-label ketamine. J Psychopharmacol. (2015) 29:596–607. 10.1177/0269881114568041 25691504 PMC5116382

[17] Zheng W Gu LM Sun CH Zhou YL Wang CY Lan XF. Comparative effectiveness of repeated ketamine infusions in treating anhedonia in bipolar and unipolar depression. J Affect Disord. (2022) 300:109–13. 10.1016/j.jad.2021.12.105 34965393

[18] Zheng W Gu LM Zhou YL Wang CY Lan XF Zhang B. Association of VEGF with antianhedonic effects of repeated-dose intravenous ketamine in treatment-refractory depression. Front Psychiatry. (2021) 12:780975. 10.3389/fpsyt.2021.780975 34925104 PMC8677831

[19] Berman RM Cappiello A Anand A Oren DA Heninger GR Charney DS. Antidepressant effects of ketamine in depressed patients. Biol Psychiatry. (2000) 47:351–4. 10.1016/S0006-3223(99)00230-9 10686270

[20] Kishimoto T Chawla JM Hagi K Zarate CA Kane JM Bauer M. Single-dose infusion ketamine and non-ketamine N-methyl-d-aspartate receptor antagonists for unipolar and bipolar depression: a meta-analysis of efficacy, safety and time trajectories. Psychol Med. (2016) 46:1459–72. 10.1017/S0033291716000064 26867988 PMC5116384

[21] Ballard ED Ionescu DF Vande Voort JL Niciu MJ Richards EM Luckenbaugh DA. Improvement in suicidal ideation after ketamine infusion: relationship to reductions in depression and anxiety. J Psychiatr Res. (2014) 58:161–6. 10.1016/j.jpsychires.2014.07.027 25169854 PMC4163501

[22] Zhan Y Zhang B Zhou Y Zheng W Liu W Wang C. A preliminary study of anti-suicidal efficacy of repeated ketamine infusions in depression with suicidal ideation. J Affect Disord. (2019) 251:205–12. 10.1016/j.jad.2019.03.071 30927581

[23] Murrough JW Perez AM Pillemer S Stern J Parides MK aan het Rot M. Rapid and longer-term antidepressant effects of repeated ketamine infusions in treatment-resistant major depression. Biol Psychiatry. (2013) 74:250–6. 10.1016/j.biopsych.2012.06.022 22840761 PMC3725185

[24] Minelli A Maffioletti E Bortolomasi M Conca A Zanardini R Rillosi L. Association between baseline serum vascular endothelial growth factor levels and response to electroconvulsive therapy. Acta Psychiatr Scand. (2014) 129:461–6. 10.1111/acps.12187 23957507

[25] Kao CF Liu YL Yu YW Yang AC Lin E Kuo PH. Gene-based analysis of genes related to neurotrophic pathway suggests association of BDNF and VEGFA with antidepressant treatment-response in depressed patients. Sci Rep. (2018) 8:6983. 10.1038/s41598-018-25529-y 29725086 PMC5934385

[26] Isung J Aeinehband S Mobarrez F Mårtensson B Nordström P Asberg M. Low vascular endothelial growth factor and interleukin-8 in cerebrospinal fluid of suicide attempters. Transl Psychiatry. (2012) 2:e196. 10.1038/tp.2012.123 23168996 PMC3565771

[27] Nowacka MM Obuchowicz E. Vascular endothelial growth factor (VEGF) and its role in the central nervous system: a new element in the neurotrophic hypothesis of antidepressant drug action. Neuropeptides. (2012) 46:1–10. 10.1016/j.npep.2011.05.005 21719103

[28] Deyama S Bang E Wohleb ES Li XY Kato T Gerhard DM. Role of neuronal VEGF signaling in the prefrontal cortex in the rapid antidepressant effects of ketamine. Am J Psychiatry. (2019) 176:388–400. 10.1176/appi.ajp.2018.17121368 30606046 PMC6494682

[29] Zheng W Zhou YL Wang CY Lan XF Zhang B Zhou SM. Association of plasma VEGF levels and the antidepressant effects of ketamine in patients with depression. Therap Adv Psychopharmacol. (2021) 11:20451253211014320. 10.1177/20451253211014320 34035893 PMC8132091

[30] McGrory CL Ryan KM Gallagher B McLoughlin DM. Vascular endothelial growth factor and pigment epithelial-derived factor in the peripheral response to ketamine. J Affect Disord. (2020) 273:380–3. 10.1016/j.jad.2020.04.013 32560932

[31] Zheng W Zhou YL Liu WJ Wang CY Zhan YN Li HQ. Investigation of medical effect of multiple ketamine infusions on patients with major depressive disorder. J Psychopharmacol. (2019) 33:494–501. 10.1177/0269881119827811 30789302

[32] Zheng W Zhou YL Liu WJ Wang CY Zhan YN Li HQ. Rapid and longer-term antidepressant effects of repeated-dose intravenous ketamine for patients with unipolar and bipolar depression. J Psychiatr Res. (2018) 106:61–8. 10.1016/j.jpsychires.2018.09.013 30278319

[33] Vande Voort JL Ballard ED Luckenbaugh DA Bernert RA Richards EM Niciu MJ. Antisuicidal response following ketamine infusion is associated with decreased nighttime wakefulness in major depressive disorder and bipolar disorder. J Clin Psychiatry. (2017) 78:1068–74. 10.4088/JCP.15m10440 27929610 PMC5641476

[34] Zhou Y Liu W Zheng W Wang C Zhan Y Lan X. Predictors of response to repeated ketamine infusions in depression with suicidal ideation: an ROC curve analysis. J Affect Disord. (2020) 264:263–71. 10.1016/j.jad.2020.01.001 32056760

[35] Hamilton M. A rating scale for depression. J Neurol Neurosurg Psychiatry. (1960) 23:56–62. 10.1136/jnnp.23.1.56 14399272 PMC495331

[36] Xie GR Shen QJ. Use of the Chinese version of the Hamilton Rating Scale for Depression in general population and patients with major depression (In Chinese). Chin J Nerv Ment Dis. (1984) 10:364.19440887

[37] Rasmussen KG Lineberry TW Galardy CW Kung S Lapid MI Palmer BA. Serial infusions of low-dose ketamine for major depression. J Psychopharmacol. (2013) 27:444–50. 10.1177/0269881113478283 23428794

[38] Cao L Jiao X Zuzga DS Liu Y Fong DM Young D. VEGF links hippocampal activity with neurogenesis, learning and memory. Nat Genet. (2004) 36:827–35. 10.1038/ng1395 15258583

[39] Storkebaum E Lambrechts D Carmeliet P. VEGF: once regarded as a specific angiogenic factor, now implicated in neuroprotection. Bioessays. (2004) 26:943–54. 10.1002/bies.20092 15351965

[40] Fournier NM Lee B Banasr M Elsayed M Duman RS. Vascular endothelial growth factor regulates adult hippocampal cell proliferation through MEK/ERK- and PI3K/Akt-dependent signaling. Neuropharmacology. (2012) 63:642–52. 10.1016/j.neuropharm.2012.04.033 22580375 PMC3392414

[41] Calvo CF Fontaine RH Soueid J Tammela T Makinen T Alfaro-Cervello C. Vascular endothelial growth factor receptor 3 directly regulates murine neurogenesis. Genes Dev. (2011) 25:831–44. 10.1101/gad.615311 21498572 PMC3078708

[42] Clark-Raymond A Halaris A. VEGF and depression: a comprehensive assessment of clinical data. J Psychiatr Res. (2013) 47:1080–7. 10.1016/j.jpsychires.2013.04.008 23684549

[43] Isung J Mobarrez F Nordström P Asberg M Jokinen J. Low plasma vascular endothelial growth factor (VEGF) associated with completed suicide. World J Biol Psychiatry. (2012) 13:468–73. 10.3109/15622975.2011.624549 22098148

[44] Deyama S Bang E Kato T Li XY Duman RS. Neurotrophic and Antidepressant Actions of Brain-Derived Neurotrophic Factor Require Vascular Endothelial Growth Factor. Biol Psychiatry. (2019) 86:143–52. 10.1016/j.biopsych.2018.12.014 30712809 PMC6597338

[45] Medeiros GC Greenstein D Kadriu B Yuan P Park LT Gould TD. Treatment of depression with ketamine does not change plasma levels of brain-derived neurotrophic factor or vascular endothelial growth factor. J Affect Disord. (2021) 280:136–9. 10.1016/j.jad.2020.11.011 33212404 PMC8194375

[46] Greene J Banasr M Lee B Warner-Schmidt J Duman RS. Vascular endothelial growth factor signaling is required for the behavioral actions of antidepressant treatment: pharmacological and cellular characterization. Neuropsychopharmacology. (2009) 34:2459–68. 10.1038/npp.2009.68 19553916 PMC3694572

[47] Sun R Li N Li T. VEGF regulates antidepressant effects of lamotrigine. Eur Neuropsychopharmacol. (2012) 22:424–30. 10.1016/j.euroneuro.2011.09.010 22033393

[48] Segi-Nishida E. Exploration of new molecular mechanisms for antidepressant actions of electroconvulsive seizure. Biol Pharm Bull. (2011) 34:939–44. 10.1248/bpb.34.939 21719995

[49] Warner-Schmidt JL Duman RS. VEGF as a potential target for therapeutic intervention in depression. Curr Opin Pharmacol. (2008) 8:14–9. 10.1016/j.coph.2007.10.013 18061540 PMC2259283

[50] Pompili M Venturini P Palermo M Stefani H Seretti ME Lamis DA. Mood disorders medications: predictors of nonadherence - review of the current literature. Expert Rev Neurother. (2013) 13:809–25. 10.1586/14737175.2013.811976 23898852

[51] Levy MJF Boulle F Steinbusch HW van den Hove DLA Kenis G Lanfumey L. Neurotrophic factors and neuroplasticity pathways in the pathophysiology and treatment of depression. Psychopharmacology. (2018) 235:2195–220. 10.1007/s00213-018-4950-4 29961124 PMC6061771

